# Family Functioning, Maternal Depression, and Adolescent Cognitive Flexibility and Its Associations with Adolescent Depression: A Cross-Sectional Study

**DOI:** 10.3390/children11010131

**Published:** 2024-01-22

**Authors:** Justyna Urbańska-Grosz, Emilia J. Sitek, Anna Pakalska, Bożena Pietraszczyk-Kędziora, Kalina Skwarska, Maciej Walkiewicz

**Affiliations:** 1 Rehabilitation Department of Child and Adolescent Psychiatry, Gdansk Health Center, 80-542 Gdansk, Poland; justyna.urbanska-grosz@gumed.edu.pl (J.U.-G.); emilia.sitek@gumed.edu.pl (E.J.S.);; 2 Laboratory of Clinical Neuropsychology, Neurolinguistics and Neuropsychotherapy, Division of Neurological and Psychiatric Nursing, Faculty of Health Sciences, Medical University of Gdansk, 80-952 Gdansk, Poland; 3 Department of Neurology, St. Adalbert Hospital, Copernicus PL, 80-462 Gdansk, Poland; 4 Division of Quality of Life Research, Department of Psychology, Faculty of Health Sciences, Medical University of Gdansk, 80-210 Gdansk, Poland

**Keywords:** depression, adolescents, family, mothers, mother-child relations, family cohesion

## Abstract

Background: This study explores family functioning and its associations with adolescent major depressive disorder (MDD), comparing its dynamics with healthy counterparts. Family functioning (cohesion, flexibility, communication, and satisfaction), maternal depressive symptoms, postpartum depression history, parental divorce, parental alcohol abuse, and the adolescents’ cognitive flexibility, are examined. The research incorporates the perspectives of both adolescents and mothers. Methods: The sample includes 63 mother-teenager dyads in the clinical group and 43 in the control group. Instruments encompass the Family Adaptability and Cohesion Evaluation Scales (FACES IV), Children’s Depression Inventory (CDI-2), Beck Depression Inventory (BDI-II), The Brixton Spatial Anticipation Test, and structured interviews. Results: Families of adolescents with MDD exhibit lower flexibility, cohesion, communication, and overall satisfaction. Depressed adolescents display reduced cognitive flexibility. Discrepancies were observed between adolescents’ and mothers’ perspectives as associated with adolescents’ MDD. Teenagers emphasized the severity of maternal depressive symptoms, while mothers highlighted the importance of family cohesion and flexibility. Conclusions: This study emphasizes a holistic strategy in addressing adolescent depression, including family-based assessment and therapy. Screening for maternal depressive symptoms is identified as valuable. Cognitive flexibility also needs to be addressed during therapy for depression in adolescence.

## 1. Introduction

Depression stands as one of the most prevalent mental health conditions observed during adolescence [1]. It is now widely recognized as a complex and multifactorial disorder characterized by affective, cognitive, and psychosocial symptoms [2]. The prevalence of severe depressive symptoms among adolescents has shown an upward trend in recent years, particularly amplified by the global impact of the COVID-19 pandemic [3,4,5]. Of note, the onset of depression during teenage years increases the likelihood of experiencing depressive episodes later in life [6]. Various factors influence adolescent mental health, including genetic and cognitive vulnerabilities, temperament, and school and peer factors, as well as stress [7,8,9,10,11]. Notably, family malfunctioning emerges as a significant risk factor [12,13,14,15].

Family systems theory provides a framework for understanding the influence of family dynamics on adolescent depression [16]. This approach highlights how families function as interconnected wholes, where each member’s behaviour affects the others [17]. Originating from the work of Murray Bowen and others, it views the family as a complex social network with reciprocal interactions that significantly impact both individual and collective well-being [18]. Effective family functioning, which includes the ability to meet needs and resolve conflicts, is central to maintaining a healthy mental state among family members. Consequently, better-functioning families tend to have members with fewer instances of depression and emotional distress [19]. The family systems theory implies that understanding and improving these family interactions can lead to better mental health outcomes for adolescents.

Olson’s framework addressing family functioning enables rating family systems along three fundamental dimensions: cohesion, flexibility, and communication [20]. Cohesion involves fostering strong emotional bonds between family members [20], and its decrease is associated with an increased risk of depression in adolescents [21,22,23,24,25]. Flexibility is the measure of the family system balance between stability and change; it enables families to cope with change and reduces the impact of negative events on youth’s mental health [20]. Effective family communication serves to reduce conflict and increase adaptability and cohesion [20], thus acting as a protective factor for adolescents’ psychological well-being [13,26,27]. Parental mental health is a crucial factor in adolescent well-being, as noted by various studies [28,29,30,31,32,33,34,35]. Adolescents with a strong family history of depression exhibit an elevated susceptibility to developing depressive symptoms [36,37,38] with maternal depression significantly increasing this risk [39,40,41]. Postpartum depression [42,43], prenatal depression [44,45], antenatal depression [46], perinatal depression [47], and paternal perinatal depression [48] potentially hold a pivotal role in the mental health outcomes in the offspring. Children born to depressed mothers tend to exhibit deficits in social, psychological, and cognitive domains, thereby encountering an elevated susceptibility to depression and other mental health disorders [7,49]. Additional family factors identified in the literature as exacerbating adolescent depression include parental alcoholism [50] and parental divorce [51].

The role of neurocognitive functioning is pivotal in understanding the emergence of suicidal behaviour among young people affected by mood disorders [52]. Depressed teens often demonstrate lowered executive functioning [53,54,55,56,57]. Cognitive models of depression propose that challenges in regulating mood might stem exactly from difficulties in executive functions, particularly inhibition and flexibility [58,59,60,61,62]. Additionally, parental depressive symptoms contribute to poor executive functions in the offspring [63,64,65].

### Aim of This Study

This study aimed at comparing family functioning between adolescents diagnosed with major depressive disorder (MDD) and their healthy counterparts. In particular, we hypothesized that the families of adolescents with MDD would exhibit lower levels of family functioning dimensions with emphasis on family flexibility as compared to families of healthy adolescents. In addition, it was predicted that mothers of adolescents with depressive symptoms would report higher levels of depressive symptoms compared to mothers of adolescents in the control group.

The secondary aim was to identify factors associated with depression in adolescents. We hypothesized that the quality of family functioning (cohesion, flexibility, communication, and satisfaction), maternal depressive symptoms, history of postpartum depression, parental divorce, and parental alcohol abuse each contribute to the occurrence of depressive symptoms in adolescents. Additionally, we included the adolescents’ cognitive flexibility, as it may be viewed both as a cognitive symptom of depression or a more general manifestation of a cognitive style modelled by a depressive state in a parent.

This study takes into consideration the perspectives of both the adolescents and their mothers.

## 2. Materials and Methods

### 2.1. Participants

The study sample consisted of mother-teenager dyads: 63 teenagers diagnosed with major depressive disorder (MDD) (56% female, aged 16.92 ± 1.3 years) and 63 mothers aged 44.98 ± 7.71 years, plus a matched control group of 43 healthy teenagers (48% female, aged 17.23 ± 1.15 years) and their mothers aged 43.54 ± 4.19 years. The study groups were rigorously matched for gender, age, and general intellectual functioning (see Table 1). The inclusion criteria were the presence of major depressive disorder according to the ICD-10 classification [66] and the age of 14–19 years. The exclusion criteria were the presence of significant somatic diseases in the child (cancer, diabetes, cystic fibrosis, etc.), other neurological conditions (e.g., epilepsy, cerebral palsy), and other mental health disorders (such as schizophrenia, eating disorders, etc.), as well as specific learning difficulties.

### 2.2. Procedure

All adolescents with MDD were outpatients at the Mental Health Clinic or Psychiatric Day Unit for Children and Adolescents of the Gdańsk Health Centre, Gdańsk, Poland. Youth from the control group were students at the Maritime School Complex in Gdańsk (Poland). All participants provided informed consent before any study procedures. For adolescents, consent was obtained from both the teenager and their mother. Teenagers completed various assessments, including the Polish version of the Family Adaptability and Cohesion Evaluation Scales (FACES IV) [67], the Children’s Depression Inventory 2 (CDI-2), and the Beck Depression Inventory-II (BDI-II) [68], and they participated in a structured interview. The general intellectual function was assessed with a Word Comprehension Test—an advanced version [69]. The Brixton Spatial Anticipation Test [70,71] served as a measure of cognitive flexibility. Mothers also completed FACES IV, assessed their child’s depression using CDI-2, and evaluated their own depressive symptoms through BDI-II (see: Supplementary Table S1). A structured interview was also conducted. This study was approved by the Medical University of Gdańsk Bioethics Committee [NKBBN/478/2018-2019] on 14 February 2019.

### 2.3. Measures

#### 2.3.1. Family Functioning

The Polish version of the Family Adaptability and Cohesion Evaluation Scales—FACES IV is a self-report tool designed to evaluate family functioning within the framework of the Circumplex model [72,73]. This questionnaire comprises 62 statements to which participants respond on a five-point scale, thereby elucidating the family’s level of cohesion, flexibility, and overall functioning. Furthermore, it encompasses the appraisal of family communication and the degree of satisfaction with family life [61]. FACES IV represents a comprehensive tool for the evaluation of family dynamics, characterized by satisfactory reliability, validity, and clinical utility [73]. The Polish adaptation of the FACES IV questionnaire was followed as adapted by Margasiński and presented as the Family Assessment Scale (SOR) [67]. Confirmatory factor analysis confirmed the compatibility of data derived from the Polish population with the theoretical tenets of Olson’s model. The original version of the scale demonstrates reliability coefficients ranging from 0.77 to 0.89. The Cronbach’s alpha coefficient for the Polish scales falls within the range of 0.70 to 0.93, thereby indicating a high or satisfactory degree of internal consistency. These obtained coefficients are deemed adequate for research endeavours, following the criteria established by Olson and Gorall in 2006 [73].

#### 2.3.2. Adolescents’ DepressionS

The Polish version of the Children’s Depression Inventory (CDI-2) is a 28-item self-report/informant-report questionnaire that assesses symptoms of adolescent depression. Items are rated on a 3-point Likert scale. CDI-2 presents good internal consistency (α = 0.82) and satisfactory discriminant validity [74,75].

#### 2.3.3. Maternal Depression

The Beck Depression Inventory-II (BDI-II) was used to analyse the depressive symptoms of adolescents and their mothers [76]. The BDI-II is a multiple-choice self-report inventory that may be administered to individuals aged 13 years and above. It consists of 21 items that assess the severity of depressive symptoms. Each item is a list of four statements arranged in increasing severity according to the DSM-IV criteria for depressive symptoms (e.g., hopelessness, irritability, guilt, fatigue, weight loss, etc.) [76]. The test has good one-week test-retest reliability (Pearson r = 0.93) and internal consistency α = 0.91 for the original version [76], as well as for the Polish version used in this study (0.93 and 0.95, respectively) [68].

#### 2.3.4. General Intellectual Function

The Word Comprehension Test—advanced version (TRS-Z) is a multiple-choice word comprehension scale that measures the level of crystallized intelligence through the assessment of receptive vocabulary. The task requires the identification of a synonym of a given stimulus word from a range of distractors. In the TRS-Z version, the test consists of 30 items [69]. The test was used to match the control group to the clinical group in terms of intellectual functioning.

#### 2.3.5. Adolescents’ Cognitive Flexibility

The Brixton Spatial Anticipation Test was used to assess cognitive flexibility in adolescents [70,71]. Participants are asked to predict the position of a blue circle that changes or stays the same according to a set of patterns. The pattern (rule) must be inferred from a series of previous positions. Sometimes the movement pattern changes and participants have to abandon an old concept for a new one [70]. The raw score corresponds to the number of errors. The scaled score ranges from 1 to 10, with higher scores corresponding to better cognitive flexibility.

### 2.4. Statistical Analysis

IBM SPSS Statistics 25.0 software and STATISTICA 13.3 were used. The normality of distribution was verified with the use of the Shapiro-Wilk test. Intergroup comparisons for adolescent-adolescent and mother-mother comparisons were performed with the use of the χ^2^ test (for categorical variables) and the Student’s *t*-test or the Mann–Whitney *U* test for independent variables, depending on the data distribution. Spearman rank correlation coefficient was used in the correlation analysis. Forward stepwise logistic regression was also performed. Moreover, the significance level was set at α = 0.05. Forward stepwise logistic regression was performed to verify whether family cohesion, flexibility, life satisfaction, communication, cognitive flexibility, mother’s depressive symptoms, divorce in the family, alcohol abuse in the family, and postpartum depression in mothers were associated with depression in adolescents. At first, the series of models were based on variables according to the adolescents’ point of view. The second series of models was based on family functioning assessed by the mothers. Controlled variables (covariates) were not included.

## 3. Results

Adolescents with MDD not only scored higher on depressive symptoms (z = 8.7064; *p* = 0.0001) but also had lower cognitive flexibility than their peers (z = 6.0568; *p* = 0.001) (see Table 1). Mothers of teenagers with depression also scored significantly higher on depression scales (z = 3.2475; *p* = 0.001) (see Table 2). Adolescents with MDD rated their families as functioning less well than their non-depressed peers (z = −3.9673; *p* = 000.1) (see Table 3 and Supplementary Table S3). In particular, they assessed their families as exhibiting lower levels of flexibility (z = −3.4132; *p* = 0.0006) and cohesion (z = −3.3088; *p* = 0.0009). Additionally, family communication (z = −5.1398; *p* = 0.0001) and satisfaction with family life (t(107) = −6.8303; *p* = 0.001) were significantly lower in the perception of adolescents with depression. These adolescents also perceived their families as having lower balanced cohesion (z = −3.6081; *p* = 0.0003) and balanced flexibility (z = −3.8149; *p* = 0.0001), while disengagement was rated as significantly higher.

Likewise, differences were observed in the perception of family functioning between mothers of depressed adolescents and those of healthy adolescents. Mothers from the clinical group assessed the functioning of their families as worse, noting lower levels of flexibility (z = −3.24118; *p* = 0.0011) and family communication (z = −4.3432; *p* = 0.0001) as well as satisfaction with family life. However, no significant differences were found in the perception of family cohesion (z = −0.8196; *p* = 0.4124) between mothers of depressed and healthy adolescents. Mothers of depressed teenagers also rated balanced family cohesion (z = −0.7389; *p* = 0.4599) and balanced family flexibility (z = −3.0029; *p* = 0.0026) less favourably than mothers of healthy teenagers.

Overall ratings of family functioning by the adolescent and by the mother were rather weakly correlated (r = 0.27; *p* < 0.005) (see Supplementary Table S4). Adolescent and mother scores measuring rigidity (r = 0.44; *p* < 0.005) and disengagement (r = 0.45; *p* < 0.005) were moderately correlated. There were also low correlations between mother and adolescent ratings in terms of all other specific aspects of family functioning with the exception of measures of communication, balanced cohesion, and chaotic functioning that did not correlate with each other. Additionally, the flexibility score as assessed by the mother showed low positive correlations with several aspects of family functioning rated by the adolescent: (balanced) cohesion (r = 0.33; *p* < 0.005) and (balanced) flexibility (r = 0.29; *p* < 0.005). Also, the mother’s flexibility score was negatively weakly correlated with enmeshment (r = −0.32; *p* < 0.005) as rated by the adolescent. Of note, satisfaction rating in the adolescent was also negatively moderately associated with disengagement rated (r = −0.40; *p* < 0.005) by the mother.

### Factors Associated with Depression in Adolescents

As a preparatory step to regression analysis, all observations scoring above or below three standard deviations in depression, family functioning, and cognitive flexibility were considered outliers and removed from further analyses. For all tested variables, distribution varied from the normal distribution. However, skewness was between −2 to 2 for every variable, and even between −1 to 1 for the variables used. Thus, logistic regression was run to verify the hypothesis.

Forward stepwise logistic regression was performed to verify whether family cohesion, flexibility, life satisfaction, communication, cognitive flexibility, mother’s depressive symptoms, divorce in the family, alcohol abuse in the family, and postpartum depression in mothers identify associations with depression in adolescents (see: Supplementary Table S2).

One outlier was removed from the analysis. The results are shown in Table 4.

Results from the first model indicated the goodness of fit of this model: χ^2^(8) = 9.29, *p* = 0.318. The model explained 52% (Nagelkerke *R*^2^ = 0.521) of the variance in depression in adolescents and correctly classified 79.6% of cases. Family life satisfaction was the most significant association with depression in adolescents.

In the second step, cognitive flexibility entered into the regression equation. The Hosmer and Lemeshow test confirmed the goodness of fit of this model: χ^2^(8) = 5.42; *p* = 0.712. The model was able to classify correctly 88% of participants. The explained variance of the dependent variable was 74% (Nagelkerke *R*^2^ = 0.739). Cognitive flexibility was an additional significant predictor.

The final third step of the model indicated that goodness of fit was met: χ^2^(8) = 3.29; *p* = 0.915. The model explained 80% of the variance of the dependent variable (Nagelkerke *R*^2^ = 0.804). The model correctly classified 89.8% of cases. Family life satisfaction, maternal depressive symptoms, and cognitive flexibility were associated with depression in adolescents. The odds of depression in adolescents were lowered on average by 7% for every additional unit in family life satisfaction. The results showed no difference between low versus average levels of maternal depressive symptoms. However, there is a significant difference between low versus high levels of depressive symptoms in the mother as correlated with depression in adolescents. At the high level of maternal depressive symptoms, the odds of adolescent depression are 1656% higher as compared to the low level of maternal depressive symptoms. Also, the odds of depression in adolescents on average lowered by 69% for every additional unit in cognitive flexibility. Family flexibility, cohesion, communication, divorce in the family, drinking alcohol in the family, and postpartum depression in mothers were not associated with the probability of depression in adolescents.

Next, logistic regression for identifying associations with depression in adolescents was conducted based on family cohesion, flexibility, life satisfaction, communication (assessed by mothers), cognitive flexibility, maternal depressive symptoms, divorce in the family, alcohol abuse in the family, and postpartum depression in mothers. The results are presented in Table 5.

The results analysis consisted of five steps. The first model showed a good fit to the data based on the Hosmer and Lemeshow test: χ^2^(5) = 12.67; *p* = 0.027. The model explained 43% of the variance of depression in participants (Nagelkerke *R*^2^ = 0.432), classifying correctly 75.2% of cases. Cognitive flexibility was the only significant factor.

The goodness of fit of the second model was met: χ^2^(8) = 14.45; *p* = 0.071. Both family life satisfaction assessed by the mothers and cognitive flexibility explained 52% of the variance in the dependent variable (Nagelkerke *R*^2^ = 0.515). The model was able to correctly classify 84.4% of participants.

The Hosmer and Lemeshow test in the third model was also not significant, which indicates the goodness of fit: χ^2^(8) = 10.24; *p* =.248. The third model explained 59% of the variance of depression in adolescents (Nagelkerke *R*^2^ = 0.589). In this model, 87.2% of cases were correctly classified. Not only cognitive flexibility and family life satisfaction but also maternal depressive symptoms were significant factors associated with adolescent depression.

The results of the fourth model confirmed goodness of fit: χ^2^(8) = 6.54; *p* = 0.587. The model explained 63% of the variance of depression in participants (Nagalkerke *R*^2^ = 0.628), based on cognitive flexibility, family life satisfaction, mother’s depression, and family cohesion. In this model, 85.3% of the participants were classified correctly, slightly less than in the previous model.

Finally, the Hosmer and Lemeshow goodness of fit test statistic showed a good fit for the final model: χ^2^(8) = 5.10; *p* = 0.747. The five factors together: cognitive flexibility, family life satisfaction, mother’s depression, family cohesion, and family flexibility explained 67% of the variance of the probability of depression occurrence in adolescents. The model correctly classified 87.2% of the participants. The results showed a higher probability of adolescent depression when maternal depressive symptoms were at high versus low levels. If maternal depressive symptoms are high, the odds of depression are 531% higher. The odds of depression in adolescents will be on average lowered by 90% for every additional unit in family flexibility. Also, the odds of adolescent depression are 2291% higher for every additional unit in family cohesion. Next, the odds of depression in adolescents are on average 5% lower for every additional unit in family life satisfaction. The odds of depression in adolescents are on average 44% lower for every additional unit in cognitive flexibility. The other factors were not significant, and for this reason, they did not enter the model.

## 4. Discussion

To our knowledge, this is the first study that identifies factors associated with adolescent depression from both familial and neuropsychological perspectives. Generally, the results of our study delineate that teenagers diagnosed with MDD compared to the control group assessed general family functioning as worse, with less favourable ratings pertaining to flexibility, cohesion, family communication, and overall satisfaction with family life. Furthermore, discernible cognitive rigidity was observed among adolescents with MDD, indicative of a potential neuropsychological underpinning to the manifestation of depressive symptomatology. Concurrently, the maternal counterparts of these adolescents exhibited a heightened prevalence of depressive symptoms in comparison to the mothers of their healthy peers, which is consistent with other studies [77,78,79]. These results were consistent with the research hypotheses. Moreover, referring to the second research aim, as anticipated, the quality of family functioning, maternal depressive symptoms, and adolescents’ cognitive flexibility emerge as important associations of adolescent depression. This discernible pattern of results exhibited a degree of generalizability to maternal evaluations, thereby reinforcing the robustness and consistency of the identified determinants of adolescent depression across diverse perspectives.

From the mothers’ perspective, the most important factors related to children’s depression are the low level of family cohesion, the severity of maternal depressive symptoms, low levels of family flexibility, adolescents’ cognitive flexibility, and satisfaction with family life. However, from the point of view of teenagers, the most important link with their depression is the severity of depressive symptoms in the mother, the presence of which in our model significantly increased the likelihood of depression in an adolescent. Other factors were low cognitive flexibility and satisfaction with family life. Surprisingly, postpartum depression, family history of divorce, and parental alcoholism did not turn out to be significant correlations and did not support our hypotheses.

This result seems to be consistent with other studies that perceptions of family cohesion decrease during adolescence [80,81,82]. Mothers perceive the family as more cohesive and flexible than their children [83]. Family cohesion is negatively associated with the severity of depressive symptoms in adolescents [22,23,24,25,84,85]. Family cohesion pertains to the emotional connections existing among family members and the degree of autonomy that individuals are afforded within the familial unit. Optimal conditions for individual development are thought to be achieved when a moderate level of cohesion is maintained, enabling a delicate equilibrium between autonomy and attachment. However, excessively high levels of cohesion can lead to over-identification and excessive loyalty within the family, potentially impeding the development of individualization and autonomy. Conversely, excessively low levels of cohesion may render the establishment of a coherent family community challenging [20,72,73,86]. In our study, we did not examine associations with peer relationships. Findings from other studies show the covariation of parent-friend relationship quality and adolescent depressive mood and emphasize that parent and peer effects are not independent of each other—supporting compensatory and additive models at the within-individual model and enhancing and additive models at the between-individual level. These findings highlight the robustness of the protective effects of parental and peer support and the detrimental effects of conflictual relationships on adolescent mental health [87].

The results of this study suggest that, in the opinion of the mothers, the risk of depression in their children will be lower, the greater the flexibility of the family. Flexibility focuses on how the family system balances stability and change [20], enables families to cope with change, and reduces the impact of negative events on youth mental health [84]. High family flexibility helps adolescents effectively cope with the effects of external negative life events and reduces the risk of depression [84].

A shared association of teenage depression for both mothers and adolescents resides in their respective levels of satisfaction with family life. This finding may not be particularly unexpected. However, an additional noteworthy factor related to adolescent depression, as perceived by mothers, is family flexibility, denoting the family’s adaptability in response to changing circumstances. Notably, a higher level of family flexibility is associated with adolescents’ enhanced capacity to effectively manage the repercussions of external adversities in their lives, consequently reducing the risk of depression [13,24].

Good communication reduces family conflicts and increases adaptability and cohesion [20], thus playing a protective role for adolescents’ mental health [88]. Mothers report the most psychological symptoms when adolescents and mothers agree that family functioning is poor (e.g., low open communication, high communication problems, and low family satisfaction) [80].

In our study, current maternal depressive symptoms were more important than a history of postpartum depression. As shown in Chithiramohan’s [41] review, children of mothers with postpartum depression are almost twice as likely to develop depression (in adolescence or adulthood) than children without this history. Empirical findings on childhood parental divorce and subsequent mental health outcomes are not fully consistent. Teenagers’ mental problems intensify after their parents’ divorce [51], and divorce may also be a risk factor for suicide attempts [89]. Parental divorce is currently quite a common phenomenon in families. Marital instability is not a single risk factor, but a cascade of consequences for children. Individual, family, ethnic, and cultural factors mitigate the risks associated with changes in children’s family lives, highlighting the importance of recognizing family diversity [90]. According to research, alcohol abuse in parents is associated with the occurrence of depressive disorders [91,92,93]. In our study, these factors did not prove to be significant correlates of depression in teenagers. The research results do not confirm the rather simplistic but often widespread view of the inevitable “psychopathology” in the offspring of people addicted to alcohol [94,95].

Generally, teenagers evaluate family functioning as worse than their parents [80,82]. The fact that in our study the adolescents’ and mothers’ perspectives are not fully consistent (e.g., the significance of maternal depressive symptoms vs. family cohesion) is interesting. We assume that it may be related to the developmental phase in which adolescents experience a pronounced inclination to gradually distance themselves from their familial units, intensifying their focus on peer relationships. Simultaneously, adolescents desire independence; however, from their perspective, maternal depression symptoms may hinder this process. This situation can elicit a myriad of emotional responses, including sentiments of guilt for seemingly “abandoning” depressive mothers. Conversely, the mother may attribute her child’s depression to the perception that the teenager is “growing apart” from the family unit. She may endeavour to provide support and draw closer to her child, although the teenager’s requirements for such proximity may have evolved. The mother might not fully discern this evolving dynamic. Of note, this is in line with the clinical observations of how when faced with their teenagers’ depressive symptoms, mothers as their primary response frequently seek to foster familial togetherness. This inclination to define the adolescent’s emotional detachment from the family as the root cause of depression leads them to believe that nurturing emotional closeness represents the optimal means of supporting their children. This approach, however, appears counterproductive when viewed within the context of the natural developmental needs of adolescents. Moreover, mothers, conceivably burdened by guilt associated with perceiving a growing familial rift, regard this natural developmental trajectory as an aberration and strive, albeit unsuccessfully, to counteract it, experiencing depressive reactions. While high maternal engagement may prevent depression in adolescents [96], high engagement of depressive mothers may act in the opposite direction. Individuation has a positive prognostic value in adolescent depression [97]. Also, the discrepancy may be related to a more general phenomenon of dissimulation—parents usually tend to present their parental practice as better than it is in reality [98]. This pattern of responding could affect ratings of family functioning as well. Of note, in a novel analysis using polynomial regression, it was shown that discrepancies in father-child relationship assessment were more strongly related to depressive symptoms in early adolescence than those in mother-child relationship assessment [99]. We assessed differences in mothers’ and adolescents’ perceptions of functioning as suggested by the recommendation to use regression analysis rather than using difference scores as hypothesis tests with informant discrepancies [81,100]. Recent work indicates that differences may exist in terms of family functioning [101,102,103]. Not all teenagers and parents differ in their perception of family functioning. In samples of adolescent–parent dyads, some assessments of family functioning are quite consistent with each other, while others are not [101,104]. Sometimes this is because the parent perceives the family as functioning more favourably than the teenager, and sometimes the opposite is true: the teenager perceives the family more favourably than the parent [105,106,107].

Adolescents’ cognitive flexibility encompasses the aptitude to apprehend situations from diverse spatial or interpersonal vantage points, to transition seamlessly between tasks, and to adapt their responses in accordance with changing circumstances [108]. This attribute assumes a protective role against depression, particularly among individuals whose parents have encountered depressive experiences [15]. Adolescent cognitive flexibility emerges as a valuable asset for young individuals grappling with the tribulations of parental depression, as elucidated by Davidovich et al. in 2016 [62]. This becomes especially salient given that, within our study, mothers of teenagers diagnosed with MDD registered notably higher scores on depressiveness scales. Longitudinal studies highlight a strong association between parental depressive symptoms and executive functioning in young people [63,64,65]. Neurocognitive functioning plays a pivotal role in the manifestation of suicidal behaviour in young people with affective disorders [52]. Cognitive models of depression propose that the challenges in mood regulation may stem from difficulties with executive functions such as inhibition and flexibility [58,59,60,61,109]. It is also worth noting that higher levels of inhibition and flexibility in the offspring of depressed parents are associated with lower symptoms of depression in adolescents [62]. The relationship between cognitive flexibility and depression seems multifaceted. Of course, cognitive flexibility in MDD may be viewed as a cognitive deficit related to depression. However, we argue that it may also be a sign of mental rigidity associated with family inflexibility. There is evidence that affective parenting behaviour may impact brain development in children, since high aggression and low positivity are associated with depression and suboptimal brain development in children [110]. To fully appreciate the emergence of cognitive flexibility deficits and their neurobiological and/or familial origin in adolescents with MDD, a prospective study would be needed, starting from a large population-based neuropsychological and neuroimaging study and then comparing the subset of adolescents who developed MDD and those who did not. This study design seems unfeasible not only due to its complexity but also the difficulty in longitudinal measurement of executive function due to practice effects [70]. There are very few measures of cognitive flexibility that have alternate versions, and if so, only two versions are available. Although the Brixton Spatial Anticipation Test was shown to be quite resistant to practice effects in one study [111], the executive task is not novel if used for the second time and its use in longitudinal assessment warrants caution. While teenagers’ cognitive functioning, encompassing cognitive flexibility, might not intuitively appear closely related to family dynamics or maternal depression, our study’s findings suggest its considerable predictive potential. This dimension of cognitive flexibility could be instrumental in shaping perceptions of family functioning and might indirectly account for the disparities in the assessment of factors related to depressive disorders between mothers and adolescents. Notably, it is worth emphasizing that our study did not scrutinize the cognitive flexibility of mothers, but this avenue presents an intriguing prospect for future investigations.

Within the framework of systemic theory, a foundational assumption lies in the recognition that family members can potentially disrupt the trajectory of adolescent independence [112]. This interference, it is posited, emanates from the perceived threat it poses to the equilibrium, or homeostasis, of the family system. In a complex and intricate way, adolescents reacting to family disruption may assume a role in preserving familial homeostasis by manifesting depressive symptoms. The systemic approach seeks to identify the origins of disorders not within the individual but within the realm of family interactions. This perspective underscores the pivotal influence of these familial dynamics on the individual’s developmental trajectory [113,114]. In essence, the systemic approach refutes the notion of individual pathology, instead positing that interdependent (and potentially atypical) family interactions may serve as a pathogenic element in the emergence of disorders within one or more family members [115].

As shown by our study, family factors have a crucial importance for adolescent depression, and thus a systemic approach to treatment seems optimal. Individual psychotherapy is unlikely to modify the family dynamics. Until now, however, the literature on the efficacity of family-based interventions is scarce [116], and only a small positive effect of involving family/caregivers in the therapy was proven [117]. Of note, most of the studies documenting the effects of family-based interventions used cognitive-behavioural therapy approaches, while attachment-based interventions show particular promise in repairing parent-child communication [118,119].

### Limitations of This Study and Future Research

One limitation of our study is that we did not assess the level of depressive symptoms in fathers. This is due to the probably poorer engagement of fathers in children’s mental health treatment. Mothers tend to more actively participate in the psychological diagnostic process. An interesting avenue for further exploration could involve examining family functioning from the perspectives of other family members, such as fathers and siblings. Notably, paternal depression can be a factor related with depressive symptoms in children to an equal or even greater extent than maternal depression [9,120]. Apart from maternal anxiety and depression, emotional problems in the father are important correlates of depression in late adolescence [31]. Other findings highlight the father’s role in promoting resilience in maternal depression and underscore the need for father-focused interventions [121].

Expanding our study to include data on fathers could yield valuable insights into their level of depressive symptoms, their views on family functioning, their involvement, and whether they contribute to buffering family dynamics or possess other mental health issues. Additionally, examining peer relations and including data from other family members could be worth broader analyses. Factors contributing to resilience are multifaceted, encompassing elements within the individual, the family, and the broader social environment [122]. This suggests the importance of considering this factor more comprehensively in future research endeavours.

Another limitation is that we did not assess maternal cognitive flexibility, which may have also yielded valuable insights. The literature indicates that deficits in cognitive flexibility in mothers may be linked to a child’s depression [64,123]. The cognitive deficits observed in depressed adults are crucial in the context of parenting [124,125], including the notion that depressed parents may struggle with the cognitive flexibility needed to adapt to their child’s evolving needs [124]. Considering the investigation of cognitive flexibility in depressed mothers may be a potential avenue for future research.

Religion has been identified as a protective factor in parent-child relationships, offering support in the face of mental health adversities [126]. This seems to be an intriguing research perspective for future exploration.

Comparative studies across different cultural contexts can provide valuable insights into the universality or cultural specificity of the identified determinants.

## 5. Conclusions

Families of adolescents with MDD were found to exhibit lower levels of flexibility, cohesion, family communication, and overall satisfaction with family life compared to healthy adolescents. Adolescents with MDD showed reduced cognitive flexibility. Mothers of adolescents with depressive symptoms reported higher levels of depressive symptoms compared to mothers of healthy adolescents. Quality of family functioning, maternal depressive symptoms, and adolescent cognitive flexibility were identified as important factors associated with adolescent depression. Discrepancies were observed between the perspectives of adolescents and their mothers: teenagers emphasized the severity of maternal depressive symptoms, while mothers highlighted the importance of family cohesion and flexibility. Postpartum depression, parental divorce, and parental alcoholism did not prove to be significant factors related to adolescent depression.

This study contributes to the burgeoning body of knowledge surrounding adolescent mental health, shedding light on the intricate interplay between familial dynamics, maternal mental health, and neurocognitive factors in the aetiology of depressive disorders. The results emphasize the need for a holistic strategy when addressing adolescent depression. It underscores the importance of conducting assessment and therapy that encompasses the entire family system. In particular, screening for maternal depressive symptoms emerges as a potentially valuable component of this approach. Another notable facet pertains to the heightened significance of emphasizing cognitive flexibility in the treatment efforts aimed at adolescents with depression. To date, it has received limited attention in the existing scientific literature on adolescent depression. Nonetheless, it holds considerable promise in mitigating the potential progression of depressive symptoms in this population. These findings not only underscore the necessity for multifaceted therapeutic interventions, but also argue for the integration of familial and individual-level assessments in clinical and preventive mental health paradigms.

## Figures and Tables

**Table 1:** Differences between teenagers with a major depressive disorder and their healthy peers.

	Depressed Adolescents (*n* = 63)		Healthy Controls (*n* = 46)			
					χ²	*p*
sex	35 girls (56%) 28 boys (44%)		22 girls (48%) 24 boys (52%)		−0.7907	0.4249
	Me ± IQR	min.–max.	Me ± IQR	min.–max.	intergroup difference	*p*
Age	17 (4)	15–19	17 (4)	15–19	z = −1.2844	0.1989
Overall intellectual functioning TRS-Z (1–30)	8 (6)	2–19	9 (5)	3–23	z = −0.1601	0.8728
Mood						
CDI-2 self-rating (T score: 0–79)	74 (8)	54–79	47 (16)	21–64	z = −8.7064	0.0001
CDI-2 assessed by mothers (T score: 0–79)	75 (30)	49–79	45 (37)	23–60	z = −8.64	0.0001
	$\bar{x}$ ± SD	min.–max.	$\bar{x}$ ± SD	min.–max.	intergroup difference	*p*
BDI-II (raw score: 0–63)	37 (8)	20–53	8 (9)	0–23	t = 8.8702	0.0001
Cognitive Flexibility						
	Me ± IQR		Me ± IQR			
The Brixton Spatial Anticipation Test (scaled score: 0–10)	4.57 (1.94)	1–10	6.98 (1.44)	4–10	z = 6.0568	0.001

BDI-II (The Beck Depression Inventory-II), CDI-2 (The Children’s Depression Inventory 2), IQR—interquartile range, Me—median, SD—standard deviation, TRS-Z (Word Comprehension Test—Advanced Version).

**Table 2:** Differences between mothers of depressed adolescents and mothers of healthy adolescents.

Mother	Depressed Adolescents (*n* = 63)		Healthy Controls (*n* = 46)			
	$\bar{x}$ ± SD/Me ± IQR	min.–max.	$\bar{x}$ ± SD/Me ± IQR	min.–max.	Intergroup Difference	*p*
Age	Me ± IQR 44.98 (4.71)	35–55	Me ± IQR 43.54 (4.19)	36–54	t = 1.65	0.10
BDI-II	$\bar{x}$ ± SD 15 (16)	0–43	$\bar{x}$ ± SD 8 (5)	3–25	z = 3.2475	0.0011

BDI-II (The Beck Depression Inventory-II), IQR—interquartile range, Me—median, SD—standard deviation.

**Table 3:** Assessment of family functioning.

Family Functioning	Depressed Adolescents (*n* = 63)		Healthy Controls (*n* = 46)			
A—Adolescent M—Mother	Me ± IQR	min.–max.	Me ± IQR	min.–max.	Intergroup Difference	*p*
General Family Functioning A (0–3)	0.56 ± 0.41	0.21–1.77	0.78 ± 0.42	0.40–2.00	z = −3.9673	0.0001
General Family Functioning M (0–3)	0.72 ± 0.66	0.15–1.89	0.91 ± 0.30	0.48–1.75	z = −2.2090	0.0271
Cohesion A (0–3)	0.53 ± 0.47	0.13–1.75	0.77 ± 0.46	0.24–2.50	z = −3.3088	0.0009
Cohesion M (0–3)	0.67 ± 0.70	0.13–3.00	0.77 ± 0.28	0.40–1.80	z = −0.8196	0.4124
Flexibility A (0–3)	0.63 ± 0.40	0.25–3.00	0.87 ± 0.54	0.46–2.40	z = −3.4132	0.0006
Flexiblity M (0–3)	0.77 ± 0.59	0.18–2.00	1.00 ± 0.40	0.57–2.33	z = −3.24118	0.0011
Communication raw score A (0–50)	28 ± 12	12–49	37.50 ± 11	23–50	z = −5.1398	0.0001
Communication raw score M (0–50)	35 ± 10	4–50	39 ± 7	29–48	z = −4.3432	0.0001
	$\bar{x}$ ± SD		$\bar{x}$ ± SD			
Satisfaction raw score A (0–50)	27.27 ± 6.81	13–48	38.39 ± 6.60	24–50	t = −6.8303	0.0001
Satisfaction raw score M (0–50)	32.40 ± 7.23	13–48	39.17 ± 4.94	30–47	t = −5.0082	0.0001

IQR—interquartile range, Me—median, SD—standard deviation.

**Table 4:** Models of factors associated with adolescents’ depression resulting from regression analysis with family functioning as assessed by adolescents.

Model	Factors Associated	B	SE	Wald	df	*p*	Exp(B) [95% CI]
1	Family life satisfaction (FACES IV)	−0.05	0.01	31.84	1	<0.001	0.95 [0.93–0.97]
	Intercept	2.55	0.47	29.86	1	<0.001	12.83
	Model χ^2^(8) = 9.29, *p* = 0.318; Nagelkerke *R*^2^ = 0.521						
2	Family life satisfaction (FACES IV)	−0.06	0.01	20.79	1	<0.001	0.94 [0.91–0.97]
	Cognitive flexibility (The Brixton Spatial Anticipation Test)	−1.15	0.29	15.49	1	<0.001	0.32 [0.18–0.56]
	Intercept	9.90	2.22	19.82	1	<0.001	19,862.73
	Model χ^2^(8) = 5.42; *p* = 0.712; Nagelkerke *R*^2^ = 0.739						
3	Family life satisfaction (FACES IV)	−0.08	0.02	17.76	1	<0.001	0.93 [0.89–0.96]
	BDI-II: Mother’s depressive symptoms/Baseline: low level			8.99	2	0.011	
	average level	−0.11	0.84	0.02	1	0.896	0.90 [0.17–4.69]
	high level	2.87	1.09	6.91	1	0.009	17.59 [2.07–149.20]
	Cognitive flexibility (The Brixton Spatial Anticipation Test)	−1.17	0.33	12.66	1	<0.001	0.31 [0.16–0.59]
	Intercept	9.84	2.51	15.33	1	<0.001	18,850.75
	Model χ^2^(8) = 3.29; *p* = 0.915; Nagelkerke *R*^2^ = 0.804						

BDI-II (The Beck Depression Inventory-II), FACES IV (Family Adaptability and Cohesion Evaluation Scales).

**Table 5:** Models of factors associated with depression resulting from the stepwise forward regression analysis with family functioning assessed by the mothers.

Model	Factors Associated	B	SE	Wald	df	*p*	Exp(B) [95% CI]
1	Cognitive flexibility (The Brixton Spatial Anticipation Test)	−0.82	0.17	24.21	1	<0.001	0.44 [0.32–0.61]
	Intercept	5.10	1.03	24.55	1	<0.001	163.99
2	Family life satisfaction (FACES IV)	−0.03	0.01	9.27	1	0.002	0.97 [0.95–0.99]
	Cognitive flexibility (The Brixton Spatial Anticipation Test)	−0.76	0.18	17.87	1	<0.001	0.47 [0.33–0.67]
	Intercept	6.20	1.24	25.03	1	<0.001	491.56
3	Mother’s depression/Baseline: low level			8.66	2	0.013	
	average level BDI-II:	−0.10	0.62	0.02	1	0.877	0.91 [0.27–3.04]
	high level	1.95	0.73	7.15	1	0.007	7.03 [1.68–29.37]
	Family life satisfaction (FACES IV)	−0.03	0.01	9.63	1	0.002	0.97 [0.95–0.99]
	Cognitive flexibility (The Brixton Spatial Anticipation Test)	−0.67	0.18	14.01	1	<0.001	0.51 [0.36–0.73]
	Intercept	5.35	1.28	17.57	1	<0.001	210.73
4	Mother’s depression/Baseline: low level			7.04	2	0.030	
	average level BDI-II:	0.05	0.64	0.01	1	0.943	1.05 [0.30–3.68]
	high level	1.86	0.75	6.10	1	0.013	6.45 [1.47–28.30]
	Family cohesion (FACES IV)	2.11	0.96	4.82	1	0.028	8.21 [1.25–53.72]
	Family life satisfaction (FACES IV)	−0.05	0.01	12.21	1	<0.001	0.95 [0.93–0.98]
	Cognitive flexibility (The Brixton Spatial Anticipation Test)	−0.65	0.18	12.34	1	<0.001	0.52 [0.36–0.75]
	Intercept	4.22	1.35	9.80	1	0.002	67.71
5	Mother’s depression/Baseline: low level			6.01	2	0.049	
	average level BDI-II:	0.04	0.67	0.00	1	0.952	1.04 [0.28–3.89]
	high level	1.84	0.80	5.31	1	0.021	6.31 [1.32–30.19]
	Family flexibility (FACES IV)	−2.27	1.07	4.47	1	0.034	0.10 [0.01–0.85]
	Family cohesion (FACES IV)	3.17	1.17	7.31	1	0.007	23.91 [2.39–238.77]
	Family life satisfaction (FACES IV)	−0.05	0.01	11.92	1	0.001	0.95 [0.92–0.98]
	Cognitive flexibility (The Brixton Spatial Anticipation Test)	−0.57	0.19	9.06	1	0.003	0.56 [0.39–0.82]
	Intercept	5.14	1.47	12.17	1	<0.001	171.23

BDI-II (The Beck Depression Inventory-II), FACES IV (Family Adaptability and Cohesion Evaluation Scales).

## Data Availability

The data presented in this study are available on request from the corresponding author. The data are not publicly available due to the imperative to uphold patient confidentiality, especially for those undergoing psychiatric treatment.

## Supplementary Materials

The following supporting information can be downloaded at https://www.mdpi.com/article/10.3390/children11010131/s1. Table S1. Research methods used in the study. Table S2. Differences between teenagers with a major depressive disorder and their healthy peers. Table S3. Other aspects of family functioning. Table S4. Correlation analysis between the results of family functioning in the assessment of a teenager (A) and a mother (M).

## References

[1] Mullen S. (2018). Major Depressive Disorder in Children and Adolescents. Ment. Health Clin..

[2] Guerrera C.S., Platania G.A., Boccaccio F.M., Sarti P., Varrasi S., Colliva C., Grasso M., De Vivo S., Cavallaro D., Tascedda F. (2023). The Dynamic Interaction between Symptoms and Pharmacological Treatment in Patients with Major Depressive Disorder: The Role of Network Intervention Analysis. BMC Psychiatry.

[3] Hawes M.T., Szenczy A.K., Klein D.N., Hajcak G., Nelson B.D. (2022). Increases in Depression and Anxiety Symptoms in Adolescents and Young Adults during the COVID-19 Pandemic. Psychol. Med..

[4] Shorey S., Ng E.D., Wong C.H.J. (2022). Global Prevalence of Depression and Elevated Depressive Symptoms among Adolescents: A Systematic Review and Meta-Analysis. Br. J. Clin. Psychol..

[5] Courtney D., Watson P., Battaglia M., Mulsant B.H., Szatmari P. (2020). COVID-19 Impacts on Child and Youth Anxiety and Depression: Challenges and Opportunities. Can. J. Psychiatry.

[6] Johnson D., Dupuis G., Piche J., Clayborne Z., Colman I. (2018). Adult Mental Health Outcomes of Adolescent Depression: A Systematic Review. Depress. Anxiety.

[7] Garber J., Cole D.A. (2010). Intergenerational Transmission of Depression: A Launch and Grow Model of Change across Adolescence. Dev. Psychopathol..

[8] Malhotra S., Sahoo S. (2018). Antecedents of Depression in Children and Adolescents. Ind. Psychiatry J..

[9] Shore L., Toumbourou J.W., Lewis A.J., Kremer P. (2018). Review: Longitudinal Trajectories of Child and Adolescent Depressive Symptoms and Their Predictors—A Systematic Review and Meta-Analysis. Child Adolesc. Ment. Health.

[10] Fiorilli C., Capitello T.G., Barni D., Buonomo I., Gentile S. (2019). Predicting Adolescent Depression: The Interrelated Roles of Self-Esteem and Interpersonal Stressors. Front. Psychol..

[11] Pedersen G.A., Lam C., Hoffmann M., Zajkowska Z., Walsh A., Kieling C., Mondelli V., Fisher H.L., Gautam K., Kohrt B.A. (2023). Psychological and Contextual Risk Factors for First-Onset Depression among Adolescents and Young People around the Globe: A Systematic Review and Meta-Analysis. Early Interv. Psychiatry.

[12] Şırelı Ö., Aysev Soykan A. (2016). Depresyonu Olan Ergenlerin Anne-Baba Kabul-Red Algıları ve Aile Işlevleri Açısından Incelenmesi. Anadolu Psikiyatr. Derg..

[13] Guerrero-Muñoz D., Salazar D., Constain V., Perez A., Pineda-Cañar C.A., García-Perdomo H.A. (2021). Association between Family Functionality and Depression: A Systematic Review and Meta-Analysis. Korean J. Fam. Med..

[14] Huang X., Hu N., Yao Z., Peng B. (2022). Family Functioning and Adolescent Depression: A Moderated Mediation Model of Self-Esteem and Peer Relationships. Front. Psychol..

[15] Stevens E.S., Funkhouser C.J., Auerbach R.P., Talati A., Gameroff M.G., Posner J.E., Weissman M.M., Shankman S.A. (2023). Inhibition Predicts the Course of Depression and Anxiety Symptoms Among Adolescents: The Moderating Role of Familial Risk. J. Nerv. Ment. Dis..

[16] Becvar R.J., Stroh-Becvar D., Reif L.V. (2023). Systems Theory and Family Therapy.

[17] Cox M.J., Paley B. (1997). Families as systems. Annu. Rev. Psychol..

[18] Haefner J. (2014). An Application of Bowen Family Systems Theory. Issues Ment. Health Nurs..

[19] Beavers R., Hampson R.B. (2000). The Beavers Systems Model of Family Functioning. J. Fam. Ther..

[20] Olson D.H., Waldvogel L., Schlieff M. (2019). Circumplex Model of Marital and Family Systems: An Update. J. Fam. Theory Rev..

[21] Reinherz H.Z., Paradis A.D., Giaconia R.M., Stashwick C.K., Fitzmaurice G. (2003). Childhood and Adolescent Predictors of Major Depression in the Transition to Adulthood. Am. J. Psychiatry.

[22] Roley M.E., Kawakami R., Baker J., Hurtado G., Chin A., Hovey J.D. (2014). Family Cohesion Moderates the Relationship between Acculturative Stress and Depression in Japanese Adolescent Temporary Residents. J. Immigr. Minor. Health.

[23] Jhang F.H. (2017). Economically Disadvantaged Adolescents’ Self-Concept and Academic Achievement as Mediators between Family Cohesion and Mental Health in Taiwan. Int. J. Ment. Health Addict..

[24] Láng A. (2018). Family Structure, Family Functioning, and Well-Being in Adolescence: A Multidimensional Approach. Int. J. Humanit. Soc. Sci..

[25] Zahra S.T., Saleem S. (2021). Family Cohesion and Depression in Adolescents: A Mediating Role of Self-Confidence. J. Pak. Med. Assoc..

[26] Díez-Goñi N., Rodríguez-Díez M.C. (2017). Why Teaching Empathy Is Important for the Medical Degree. Rev. Clin. Esp..

[27] Li M., Li L., Wu F., Cao Y., Zhang H., Li X., Zou J., Guo Z., Kong L. (2021). Perceived Family Adaptability and Cohesion and Depressive Symptoms: A Comparison of Adolescents and Parents during COVID-19 Pandemic. J. Affect. Disord..

[28] Collishaw S., Hammerton G., Mahedy L., Sellers R., Owen M.J., Craddock N., Thapar A.K., Harold G.T., Rice F., Thapar A. (2016). Mental Health Resilience in the Adolescent Offspring of Parents with Depression: A Prospective Longitudinal Study. Lancet Psychiatry.

[29] Yeh Z.T., Huang Y.H., Liu S.I. (2016). Maternal Depression and Adolescent Emotions: The Role of Family Functioning. J. Child Fam. Stud..

[30] Weissman M.M. (2016). Children of Depressed Parents—A Public Health Opportunity. JAMA Psychiatry.

[31] Ayano G., Betts K., Lin A., Tait R., Alati R. (2021). Maternal and Paternal Mental Health Problems and the Risk of Offspring Depression in Late Adolescence: Findings from the Raine Study. J. Ment. Health.

[32] Griffith J.M., Young J.F., Hankin B.L. (2021). Longitudinal Coupling of Depression in Parent-Adolescent Dyads: Within- and Between-Dyad Effects over Time. Clin. Psychol. Sci. J. Assoc. Psychol. Sci..

[33] McAdams T.A., Rijsdijk F.V., Neiderhiser J.M., Narusyte J., Shaw D.S., Natsuaki M.N., Spotts E.L., Ganiban J.M., Reiss D., Leve L.D. (2015). The Relationship between Parental Depressive Symptoms and Offspring: Evidence from a Children-of-Twins Study and an Adoption Study. Psychol. Med..

[34] Coles D.C., Cage J. (2022). Mothers and Their Children: An Exploration of the Relationship Between Maternal Mental Health and Child Well-Being. Matern. Child Health J..

[35] Agnafors S., Sydsjö G., Svedin C.G., Bladh M. (2023). Symptoms of Depression and Internalizing Problems in Early Adulthood–Associated Factors from Birth to Adolescence. Nord. J. Psychiatry.

[36] Daches S., Vine V., Layendecker K.M., George C.J., Kovacs M. (2018). Family Functioning as Perceived by Parents and Young Offspring at High and Low Risk for Depression. J. Affect. Disord..

[37] Elsayed N.M., Fields K.M., Olvera R.L., Williamson D.E. (2019). The Role of Familial Risk, Parental Psychopathology, and Stress for First-Onset Depression during Adolescence. J. Affect. Disord..

[38] Van Dijk M.T., Murphy E., Posner J.E., Talati A., Weissman M.M. (2021). Association of Multigenerational Family History of Depression With Lifetime Depressive and Other Psychiatric Disorders in Children: Results from the Adolescent Brain Cognitive Development (ABCD) Study. JAMA Psychiatry.

[39] Mennen F.E., Negriff S., Schneiderman J.U., Trickett P.K. (2018). Longitudinal Associations of Maternal Depression and Adolescents’ Depression and Behaviors: Moderation by Maltreatment and Sex. J. Fam. Psychol..

[40] Xerxa Y., Rescorla L.A., van der Ende J., Hillegers M.H.J., Verhulst F.C., Tiemeier H. (2021). From Parent to Child to Parent: Associations Between Parent and Offspring Psychopathology. Child Dev..

[41] Chithiramohan T., Eslick G.D. (2023). Association Between Maternal Postnatal Depression and Offspring Anxiety and Depression in Adolescence and Young Adulthood: A Meta-Analysis. J. Dev. Behav. Pediatr..

[42] Sanger C., Iles J.E., Andrew C.S., Ramchandani P.G. (2015). Associations between Postnatal Maternal Depression and Psychological Outcomes in Adolescent Offspring: A Systematic Review. Arch. Womens Ment. Health.

[43] Kwong A.S.F., López-López J.A., Hammerton G., Manley D., Timpson N.J., Leckie G., Pearson R.M. (2019). Genetic and Environmental Risk Factors Associated With Trajectories of Depression Symptoms From Adolescence to Young Adulthood. JAMA Netw. Open.

[44] Pearson R.M., Evans J., Kounali D., Lewis G., Heron J., Ramchandani P.G., O’Connor T.G., Stein A. (2013). Maternal Depression during Pregnancy and the Postnatal Period: Risks and Possible Mechanisms for Offspring Depression at 18 Years. JAMA Psychiatry.

[45] Betts K.S., Williams G.M., Najman J.M., Alati R. (2015). The Relationship between Maternal Depressive, Anxious, and Stress Symptoms during Pregnancy and Adult Offspring Behavioral and Emotional Problems. Depress. Anxiety.

[46] Waters C.S., Hay D.F., Simmonds J.R., van Goozen S.H.M. (2014). Antenatal Depression and Children’s Developmental Outcomes: Potential Mechanisms and Treatment Options. Eur. Child Adolesc. Psychiatry.

[47] Tirumalaraju V., Suchting R., Evans J., Goetzl L., Refuerzo J., Neumann A., Anand D., Ravikumar R., Green C.E., Cowen P.J. (2020). Risk of Depression in the Adolescent and Adult Offspring of Mothers With Perinatal Depression: A Systematic Review and Meta-Analysis. JAMA Netw. Open.

[48] Chen J., Zhao J., Chen X., Zou Z., Zhao N., Chen C.X. (2023). Paternal Perinatal Depression: A Concept Analysis. Nurs. Open.

[49] Lew D., Xian H., Loux T., Shacham E., Scharff D. (2022). The Longitudinal Impact of Maternal Depression and Neighborhood Social Context on Adolescent Mental Health. Front. Pediatr..

[50] Mahedy L., Hammerton G., Teyhan A., Edwards A.C., Kendler K.S., Moore S.C., Hickman M., MacLeod J., Heron J. (2017). Parental Alcohol Use and Risk of Behavioral and Emotional Problems in Offspring. PLoS ONE.

[51] Tullius J.M., De Kroon M.L.A., Almansa J., Reijneveld S.A. (2022). Adolescents’ Mental Health Problems Increase after Parental Divorce, Not before, and Persist until Adulthood: A Longitudinal TRAILS Study. Eur. Child Adolesc. Psychiatry.

[52] McHugh C.M., Iorfino F., Crouse J.J., Tickell A., Nichles A., Zmicerevska N., Ho N., Lee R., Hermens D.F., Scott E. (2021). Neurocognitive Functioning Predicts Suicidal Behaviour in Young People with Affective Disorders. J. Affect. Disord..

[53] Vilgis V., Silk T.J., Vance A. (2015). Executive Function and Attention in Children and Adolescents with Depressive Disorders: A Systematic Review. Eur. Child Adolesc. Psychiatry.

[54] Wagner S., Müller C., Helmreich I., Huss M., Tadić A. (2015). A Meta-Analysis of Cognitive Functions in Children and Adolescents with Major Depressive Disorder. Eur. Child Adolesc. Psychiatry.

[55] Oliver A., Pile V., Elm D., Lau J.Y.F. (2019). The Cognitive Neuropsychology of Depression in Adolescents. Curr. Behav. Neurosci. Rep..

[56] Gillespie M.L., Rao U. (2022). Relationships between Depression and Executive Functioning in Adolescents: The Moderating Role of Unpredictable Home Environment. J. Child Fam. Stud..

[57] Urbańska-Grosz J., Walkiewicz M., Sitek E.J. (2023). Is There Sufficient Evidence for the Association between Executive Dysfunction and Academic Performance in Adolescents with Major Depressive Disorder?: A Systematic Review. Eur. Child Adolesc. Psychiatry.

[58] de Lissnyder E., Koster E.H.W., Derakshan N., de Raedt R. (2010). The Association between Depressive Symptoms and Executive Control Impairments in Response to Emotional and Non-Emotional Information. Cogn. Emot..

[59] Roiser J.P., Elliott R., Sahakian B.J. (2011). Cognitive Mechanisms of Treatment in Depression. Neuropsychopharmacology.

[60] Stange J.P., Connolly S.L., Burke T.A., Hamilton J.L., Hamlat E.J., Abramson L.Y., Alloy L.B. (2016). Inflexible Cognition Predicts First Onset of Major Depressive Episodes in Adolescence. Depress. Anxiety.

[61] Stange J.P., Alloy L.B., Fresco D.M. (2017). Inflexibility as a Vulnerability to Depression: A Systematic Qualitative Review. Clin. Psychol. Sci. Pract..

[62] Davidovich S., Collishaw S., Thapar A.K., Harold G., Thapar A., Rice F. (2016). Do Better Executive Functions Buffer the Effect of Current Parental Depression on Adolescent Depressive Symptoms?. J. Affect. Disord..

[63] Craun E., Lachance K., Williams C., Wong M.M. (2019). Parent Depressive Symptoms and Offspring Executive Functioning. J. Clin. Exp. Neuropsychol..

[64] Roman G.D., Ensor R., Hughes C. (2016). Does Executive Function Mediate the Path from Mothers’ Depressive Symptoms to Young Children’s Problem Behaviors?. J. Exp. Child Psychol..

[65] Hughes C., Roman G., Hart M.J., Ensor R. (2013). Does Maternal Depression Predict Young Children’s Executive Function?—A 4-Year Longitudinal Study. J. Child Psychol. Psychiatry.

[66] World Health Organization (2010). International Classification of Diseases.

[67] Margasiński A. (2015). The Polish Adaptation of FACES IV-SOR. Polish J. Appl. Psychol..

[68] Zawadzki B., Popiel A., Praglowska E. (2009). Psychometric Properties of the Polish Version of the Aaron T. Beck’s Depression Inventory BDI-II (Charakterystyka Psychometryczna Polskiej Adaptacji Kwestionariusza Depresji BDI-II Aarona T. Becka). Psychologia-Etologia-Genetyka.

[69] Matczak A., Jaworowska A., Martowska K. (2012). Test Rozumienia Słów: Wersja Standard TRS-S Wersja Dla Zaawansowanych TRS-Z.

[70] Burgess P.W., Alderman N., Evans J., Emslie H., Wilson B.A. (1998). The Ecological Validity of Tests of Executive Function. J. Int. Neuropsychol. Soc..

[71] Tchanturia K., Harrison A., Davies H., Roberts M., Oldershaw A., Nakazato M., Stahl D., Morris R., Schmidt U., Treasure J. (2011). Cognitive Flexibility and Clinical Severity in Eating Disorders. PLoS ONE.

[72] Olson D.H., Sprenkle D.H., Russell C.S. (1979). Circumplex Model of Marital and Family System: I. Cohesion and Adaptability Dimensions, Family Types, and Clinical Applications. Fam. Process.

[73] Olson D. (2011). FACES IV and the Circumplex Model: Validation Study. J. Marital Fam. Ther..

[74] Kovacs M., Cautin R.L., Lilienfeld S.O. (2015). Children’s Depression Inventory (CDI and CDI 2). The Encyclopedia of Clinical Psychology.

[75] Kovacs M., Wrocławska-Warchala E., Wujcik R. (2017). Zestaw Kwestionariuszy Do Diagnozy Depresji u Dzieci i Młodzieży CDI 2^TM^ Maria Kovacs i Zespół MHS: Podręcznik.

[76] Steer R.A., Ball R., Ranieri W.F., Beck A.T. (1999). Dimensions of the Beck Depression Inventory-II in Clinically Depressed Outpatients. J. Clin. Psychol..

[77] Kluczniok D., Boedeker K., Fuchs A., Hindi Attar C., Fydrich T., Fuehrer D., Dittrich K., Reck C., Winter S., Heinz A. (2016). Emotional Availability in Mother-Child Interaction: The Effects of Maternal Depression in Remission and Additional History of Childhood Abuse. Depress. Anxiety.

[78] Trussell T.M., Ward W.L., Conners Edge N.A. (2018). The Impact of Maternal Depression on Children: A Call for Maternal Depression Screening. Clin. Pediatr..

[79] Goodman S.H., Simon H.F.M., Shamblaw A.L., Kim C.Y. (2020). Parenting as a Mediator of Associations between Depression in Mothers and Children’s Functioning: A Systematic Review and Meta-Analysis. Clin. Child Fam. Psychol. Rev..

[80] Ohannessian C.M.C., Laird R., De Los Reyes A. (2016). Discrepancies in Adolescents’ and Mothers’ Perceptions of the Family and Mothers’ Psychological Symptomatology. J. Youth Adolesc..

[81] Laird R.D., De Los Reyes A. (2013). Testing Informant Discrepancies as Predictors of Early Adolescent Psychopathology: Why Difference Scores Cannot Tell You What You Want to Know and How Polynomial Regression May. J. Abnorm. Child Psychol..

[82] Steinberg L., Morris A.S. (2003). Adolescent Development. Annu. Rev. Psychol..

[83] Pérez J.C., Coo S., Irarrázaval M. (2018). Is Maternal Depression Related to Mother and Adolescent Reports of Family Functioning?☆. J. Adolesc..

[84] Nam B., Kim J.Y., DeVylder J.E., Song A. (2016). Family Functioning, Resilience, and Depression among North Korean Refugees. Psychiatry Res..

[85] Gä K., Id A., Bøe T., Breivik K., Greca A.M.L., Sivertsen B., Hysing M. (2020). Life Events and Adolescent Depressive Symptoms: Protective Factors Associated with Resilience. PLoS ONE.

[86] Olson D.H., Gorall D., Walsh F. (2003). Circumplex Model of Marital and Family Systems. Normal Family Processes.

[87] Zhang S., Baams L., van de Bongardt D., Dubas J.S. (2018). Intra- and Inter-Individual Differences in Adolescent Depressive Mood: The Role of Relationships with Parents and Friends. J. Abnorm. Child Psychol..

[88] Asarnow J.R., Tompson M.C., Klomhaus A.M., Babeva K., Langer D.A., Sugar C.A. (2020). Randomized Controlled Trial of Family-Focused Treatment for Child Depression Compared to Individual Psychotherapy: One-Year Outcomes. J. Child Psychol. Psychiatry.

[89] Donald M., Dower J., Correa-Velez I., Jones M. (2006). Risk and Protective Factors for Medically Serious Suicide Attempts: A Comparison of Hospital-Based with Population-Based Samples of Young Adults. Aust. N. Z. J. Psychiatry.

[90] D’Onofrio B., Emery R. (2019). Parental Divorce or Separation and Children’s Mental Health. World Psychiatry.

[91] Vidal S.I., Vandeleur C., Rothen S., Gholam-Rezaee M., Castelao E., Halfon O., Aubry J.M., Ferrero F., Preisig M. (2012). Risk of Mental Disorders in Children of Parents with Alcohol or Heroin Dependence: A Controlled High-Risk Study. Eur. Addict. Res..

[92] Bountress K., Chassin L. (2015). Risk for Behavior Problems in Children of Parents with Substance Use Disorders. Am. J. Orthopsychiatry.

[93] Jafry Z., Chui K., Stopka T.J., Corlin L. (2022). Residence with a Person Who Used Substances and Childhood Anxiety and Depression: A Cross-Sectional Analysis of the 2019 National Health Interview Survey. Children.

[94] Harter S.L. (2000). Psychosocial Adjustment of Adult Children of Alcoholics: A Review of the Recent Empirical Literature. Clin. Psychol. Rev..

[95] Rolf J.E., Johnson J.L., Israel E., Baldwin J., Chandra A. (1988). Depressive Affect in School-Aged Children of Alcoholics. Br. J. Addict..

[96] Jones B., Durtschi J., Keilholtz B. (2023). Maternal Engagement, Relational Closeness, and Adolescent Internalizing Symptoms: The Association of Engaged Mothering with Adolescent Depression and Anxiety. J. Marital Fam. Ther..

[97] Shi J., Tao Y., Yan C., Zhao X., Wu X., Zhang T., Zhong C., Sun J., Hu M. (2023). A Study on the Correlation between Family Dynamic Factors and Depression in Adolescents. Front. Psychiatry.

[98] Korelitz K.E., Garber J. (2016). Congruence of Parents’ and Children’s Perceptions of Parenting: A Meta-Analysis. J. Youth Adolesc..

[99] Nelemans S.A., Branje S.J.T., Hale W.W., Goossens L., Koot H.M., Oldehinkel A.J., Meeus W.H.J. (2016). Discrepancies Between Perceptions of the Parent–Adolescent Relationship and Early Adolescent Depressive Symptoms: An Illustration of Polynomial Regression Analysis. J. Youth Adolesc..

[100] Edwards J.R., Drasgow F., Schmitt N. (2002). Alternatives to Difference Scores: Polynomial Regression Analysis and Response Surface Methodology. Measuring and Analyzing Behavior in Organizations: Advances in Measurement and Data Analysis.

[101] De Los Reyes A., Ohannessian C.M.C. (2016). Introduction to the Special Issue: Discrepancies in Adolescent–Parent Perceptions of the Family and Adolescent Adjustment. J. Youth Adolesc..

[102] Lerner R.M., Konowitz L.S. (2016). Commentary: Theoretical and Methodological Dimensions of Convergence and Divergence of Adolescent and Parent Reports about Youth Development and Family Structure and Function—A Relational Developmental Systems Perspective. J. Youth Adolesc..

[103] Rote W.M., Smetana J.G. (2016). Patterns and Predictors of Mother–Adolescent Discrepancies across Family Constructs. J. Youth Adolesc..

[104] Lippold M.A., Greenberg M.T., Collins L.M. (2013). Parental Knowledge and Youth Risky Behavior: A Person Oriented Approach. J. Youth Adolesc..

[105] Lippold M.A., Greenberg M.T., Feinberg M.E. (2011). A Dyadic Approach to Understanding the Relationship of Maternal Knowledge of Youths’ Activities to Youths’ Problem Behavior Among Rural Adolescents. J. Youth Adolesc..

[106] Lippold M.A., Greenberg M.T., Collins L.M. (2014). Youths’ Substance Use and Changes in Parental Knowledge-Related Behaviors During Middle School: A Person-Oriented Approach. J. Youth Adolesc..

[107] Yaban E.H., Sayil M., Tepe Y.K. (2014). Are Discrepancies in Perceptions of Psychological Control Related to Maladjustment A Study of Adolescents and Their Parents in Turkey. Int. J. Behav. Dev..

[108] Diamond A. (2013). Executive Functions. Annu. Rev. Psychol..

[109] Harmer C.J., O’Sullivan U., Favaron E., Massey-Chase R., Ayres R., Reinecke A., Goodwin G.M., Cowen P.J. (2009). Effect of Acute Antidepressant Administration on Negative Affective Bias in Depressed Patients. Am. J. Psychiatry.

[110] Schwartz O.S., Simmons J.G., Whittle S., Byrne M.L., Yap M.B.H., Sheeber L.B., Allen N.B. (2017). Affective Parenting Behaviors, Adolescent Depression, and Brain Development: A Review of Findings From the Orygen Adolescent Development Study. Child Dev. Perspect..

[111] Burke T., Wynne B., O’Brien C., Elamin M., Bede P., Hardiman O., Pender N. (2014). Retrospective Investigations of Practice Effects on Repeated Neuropsychological Measures of Executive Functioning. Irish J. Psychol..

[112] Cox M.J., Paley B. (2003). Understanding Families as Systems. Curr. Dir. Psychol. Sci..

[113] Satir V., Ochmańska Ł., Trzebiatowska M. (2002). Rodzina: Tu Powstaje Człowiek.

[114] Cicchetti D., Rogosch F.A. (2002). A Developmental Psychopathology Perspective on Adolescence. J. Consult. Clin. Psychol..

[115] Goldenberg H., Goldenberg I., Stanton M. (2016). Family Therapy: An Overview.

[116] Eckshtain D., Horn R., Weisz J.R. (2023). Family-Based Interventions for Youth Depression: Meta-Analysis of Randomized Clinical Trials. Child Psychiatry Hum. Dev..

[117] Dippel N., Szota K., Cuijpers P., Christiansen H., Brakemeier E.L. (2022). Family Involvement in Psychotherapy for Depression in Children and Adolescents: Systematic Review and Meta-Analysis. Psychol. Psychother. Theory Res. Pract..

[118] Tsvieli N., Nir-Gottlieb O., Lifshitz C., Diamond G.S., Kobak R., Diamond G.M. (2020). Therapist Interventions Associated with Productive Emotional Processing in the Context of Attachment-Based Family Therapy for Depressed and Suicidal Adolescents. Fam. Process.

[119] Stern R.S., King A.A., Diamond G. (2023). Repairing Attachment in Families with Depressed Adolescents: A Task Analysis. J. Clin. Psychol..

[120] Tichovolsky M.H., Griffith S.F., Rolon-Arroyo B., Arnold D.H., Harvey E.A. (2018). A Longitudinal Study of Fathers’ and Young Children’s Depressive Symptoms. J. Clin. Child Adolesc. Psychol..

[121] Vakrat A., Apter-Levy Y., Feldman R. (2018). Sensitive Fathering Buffers the Effects of Chronic Maternal Depression on Child Psychopathology. Child Psychiatry Hum. Dev..

[122] Luthar S.S., Cicchetti D., Becker B. (2000). The Construct of Resilience: A Critical Evaluation and Guidelines for Future Work. Child Dev..

[123] Psychogiou L., Parry E. (2014). Why Do Depressed Individuals Have Difficulties in Their Parenting Role?. Psychol. Med..

[124] Barrett J., Fleming A.S. (2011). Annual Research Review: All Mothers Are Not Created Equal: Neural and Psychobiological Perspectives on Mothering and the Importance of Individual Differences. J. Child Psychol. Psychiatry.

[125] Bögels S.M., Lehtonen A., Restifo K. (2010). Mindful Parenting in Mental Health Care. Mindfulness.

[126] Bartkowski J.P., Xu X., Bartkowski S. (2019). Mixed Blessing: The Beneficial and Detrimental Effects of Religion on Child Development among Third-Graders. Religions.

