# Supplementary material for: Family Functioning, Maternal Depression, and Adolescent Cognitive Flexibility and Its Associations with Adolescent Depression: A Cross-Sectional Study
Source: Children (Basel). 2024 Jan 22;11(1):131. doi: 10.3390/children11010131 (PMC10814122; doi:10.3390/children11010131)
Supplement: Supplementary file 1 [file children-11-00131-s001.zip › children-2805903-supplementary.pdf]

Table S1. Research methods used in the study.

VARIABLES	METHODS	
	**adolescent**	**mother**
Adolescent' depression	BDI-II; CDI-2	CDI-2
Mother' depression	-	BDI-II
Family functioning	FACES IV	FACES IV
Adolescents' cognitive flexibility	Brixton Spatial Anticipation Test	-

Table S2. Differences between teenagers with a major depressive disorder and their healthy peers

	**Depressed adolescents (n=63)**	**Healthy controls (n=46)**	**V**	**p**
**Postpartum depression**	23 (36,50%)	6 (13,04%)	7.43	**0.0064**
**Divorce**	14 (22,22%)	8 (17,39%)	0.38	0.05367
**Alcohol abuse**	18 (28,57%)	10 (21,74%)	0.64	0.04222

Table S3. Other aspects of family functioning

**Family functioning**	**Depressed adolescents (n=63)**			**Healthy controls (n=46)**				
**A - Adolescent M - Mother**	**mean ± SD/ median±IQR**		**min.-max.**	**mean ± SD/ median±IQR**		**min.-max.**	**intergroup difference**	**p**
Balanced Cohesion raw score A (1-35)	Me±IQR	23±7	8-34	Me±IQR	27±6	16-35	z=-3.6081	**0.0003**
Balanced Cohesion raw score M (1-35)	Me±IQR	28±7	7-35	Me±IQR	28±4	23-34	z=-0.7389	0.4599
Balanced Flexibility raw score A (1-35)	Me±IQR	20±7	10-32	Me±IQR	24±6	16-34	z=--3.8149	**0.0001**
Balanced Flexibility raw score M (1-35)	Me±IQR	23±8	10-33	Me±IQR	24±5	21-33	z=--3.0029	**0.0026**
Disengaged raw score A (1-50)	x̄±SD.43	19.10±7	7-35	x̄±SD	15.57±4.43	7-26	t=-2.17049	**0.0299**
Disengaged raw score M (1-50)	x̄±SD	15.25±6.00	7-31	x̄±SD	13.52±3.31	9-22	t=1.01893	0.3082
Enmeshed raw score A (1-35)	Me±IQR	15±6	9-30	Me±IQR	14.50±6	8-23	z=-0.28629	0.7746
Enmeshed raw score M (1-35)	Me±IQR	14± 7	7-32	Me±IQR	14±5	10-20	z=--0.3725	0.7094
Rigid raw score A (1-35)	x̄±SD	17.78±4.67	7-27	x̄±SD	17.78±4.29	9-25	t=-0.1908	0.8486
Rigid raw score M (1-35)	Me±IQR	17±8	8-30	Me±IQR	17±7	1-26	z=-0.88293	0.3772
Chaotic raw score A (1-35)	Me±IQR	17±8	9-32	Me±IQR	16.50±6	8-29	z=-1.33434	0.1820
Chaotic raw score M (1-35)	Me±IQR	15±9	7-31	Me±IQR	15.50±6	7-26	z=-0.22757	0.8199

Table S4. Correlation analysis between the results of family functioning in the assessment of a teenager (A) and a mother (M).

**A - Adolescent M - mother**	**CDI-2**	**General Family Functioning A**	**Cohesion A**	**Flexibility A**	**Satisfaction A**	**Communication A**	**Balanced cohesion A**	**Balanced Flexibility A**	**Disengaged A**	**Enmeshed A**	**Rigid A**	**Chaotic A**
CDI-2		-0.21	-0.21	-0.19	-0.18	-0.07	-0.25	-0.18	0.08	0.01	0.00	0.06
BDI-II mother	0.08	-0.05	-0.08	0.00	0.08	0.08	0.00	0.09	0.08	0.19	0.19	0.08
General Family Functioning M	-0.23	**0.27***	**0.32***	0.20	0.13	0.19	**0.30***	0.23	-0.17	**-0.39***	0.00	-0.08
Cohesion M	-0.19	0.19	0.25*	0.10	0.19	0.19	0.22	0.14	-0.21	**-0.34***	0.05	-0.05
Flexibility M	-0.22	**0.32***	**0.30***	**0.29***	0.08	0.16	**0.33***	**0.29***	-0.08	**-0.32***	-0.05	-0.10
Satisfaction M	-0.18	-0.02	0.04	**-0.07**	**0.30***	**0.28***	0.00	-0.04	-0.19	-0.07	0.08	0.05
Communication M	-0.19	-0.13	-0.07	-0.15	0.24	0.11	-0.11	-0.16	-0.05	-0.11	-0.05	0.06
Balanced Cohesion M	-0.20	0.09	0.12	0.05	0.00	0.01	0.16	0.14	-0.06	-0.16	0.15	-0.05
Balanced Flexibility M	-0.20	**0.29***	**0.31***	0.22	0.08	0.15	**0.29***	**0.25***	-0.15	**-0.29***	0.04	-0.09
Disengaged M	0.17	-0.23	**-0.37***	-0.07	**-0.40***	**-0.36***	-0.24	-0.13	**0.45***	**0.27***	-0.08	-0.02
Enmeshed M	0.00	-0.20	-0.16	-0.21	-0.10	-0.08	-0.16	-0.04	0.09	**0.38***	0.34	0.16
Rigid M	-0.18	-0.09	0.00	-0.18	-0.04	-0.03	-0.04	-0.02	-0.13	0.08	**0.44***	-0.10
Chaotic M	0.29	-0.17	-0.15	**-0.26***	0.00	-0.07	-0.16	-0.23	0.04	0.18	-0.05	0.20

*p<0.005

BDI-II (The Beck Depression Inventory-II)

CDI-2 (The Children's Depression Inventory 2)
